# Bismuth encephalopathy- a rare complication of long-standing use of bismuth subsalicylate

**DOI:** 10.1186/s12883-019-1437-9

**Published:** 2019-08-29

**Authors:** Cláudia Borbinha, Filipa Serrazina, Manuel Salavisa, Miguel Viana-Baptista

**Affiliations:** 1 Neurology Department. Hospital Egas Moniz, Centro Hospitalar de Lisboa Ocidental, Lisbon, Portugal; 2 CEDOC, NOVA Medical School / Faculdade de Ciências Médicas, Lisbon, Portugal

**Keywords:** Bismuth subsalicylate, Neurotoxicity, Encephalopathy, Myoclonus

## Abstract

**Background:**

Drugs containing bismuth, although usually safe, may rarely cause neurotoxicity.

**Case presentation:**

We describe the case of a 44-year-old woman treated with bismuth subsalicylate for about 20 years, who developed abnormal behaviour and postural instability in two weeks. On examination, she had greyish discoloration of teeth, was confused and presented generalized myoclonic jerks. In the next days, her clinical condition deteriorated, with a reduction in alertness and more exuberant myoclonus. Brain MRI was unremarkable. CSF revealed mild elevation of protein content (47 mg/dL; reference range: 15-45 mg/dL) and elevation of white blood cell count (10/μL). Bismuth levels in urine (375 μg/L), serum (260 μg/L) and CSF (21.4 μg/L) samples were highly above the threshold for toxicity. Following supportive treatment and bismuth discontinuation, she made a full recovery within weeks.

**Conclusions:**

Although rare, bismuth encephalopathy should be considered in patients presenting with subacute encephalopathy and myoclonus. This encephalopathy can be subacute even after a chronic exposure. Cessation of bismuth can lead to a complete resolution in weeks.

## Background

Drugs containing bismuth have been used for many years to treat gastroenterological complaints. In addition to the efficacy in treating *H. pylori* infection, bismuth compounds also have broad anti-microbial, anti-leishmanial and anti-cancer properties [1]. Although usually safe and well tolerated, on long-standing use bismuth salts can cause a reversible syndrome characterized by subacute progression of encephalopathy, myoclonus, lack of coordination, dysarthria, seizures, parkinsonism, and neuropsychiatric symptoms [2–4]. Bismuth subsalicylate (Pepto-Bismol®) appears less likely to cause neurotoxicity than other bismuth compounds (namely bismuth subnitrate or subgallate) [2–6].

### Case presentation

A 44-year-old woman presented to the emergency department with a two-week history of abnormal behaviour, decreased concentration and postural instability. Her parents stated that she was a professionally active woman who was going through a moment of stress due to divorce. She had a past medical history of insomnia and irritable bowel syndrome and was currently being treated with vitamin D3, vitamin K, manganese and activated charcoal. Upon initial questioning, the patient and her parents denied any other medication. The patient was evaluated by her GP before hospital admission and started on lorazepam without improvement.

**Fig. 1:**
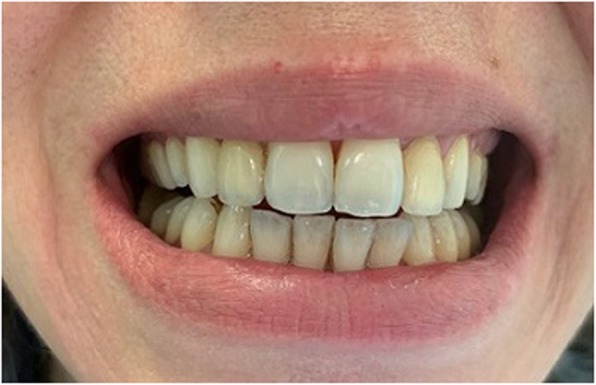
Greyish discoloration of teeth

On presentation, vital signs were normal, and general examination was notable for a greyish discoloration of teeth (Fig. 1). On neurologic examination, she was confused with impaired attention and could not express orientation correctly. Muscle tone and strength were normal, and no pyramidal signs were present. Coordination, sensation and cranial nerves were within the norms.

During her stay in the emergency department, the patient became more somnolent and began to experience more frequent myoclonic jerks. Laboratory tests, including complete blood count, renal, hepatic and thyroid functions, C-reactive protein, vitamin B12 and folate levels were within normal limits, and syphilis and HIV were negative. A noncontrast brain CT scan obtained at admission was unremarkable. A lumbar puncture showed mild elevation of protein content (47 mg/dL; reference range: 15-45 mg/dL) and elevation of white blood cell count (10/μL; normal: < 5/μL) in the CSF. Emergent treatment with IV acyclovir (10 mg/Kg IV every 8 h) and ceftriaxone (4 g/d) was started, considering a possible infectious encephalitis.

The patient was admitted to the Neurology Department, where she maintained an altered state of consciousness with a moderate reduction in alertness and exuberant myoclonus. Myoclonus was present both at rest and on action, being more severe on purposeful movements, with a generalized distribution. EEG revealed generalized background slowing indicative of diffuse and nonspecific cerebral dysfunction. Due to the patient’s lack of collaboration and the need for general anesthesia, brain MRI was postponed until the fifth day after admission, but no abnormalities were reported at that time.

Since the patient was clinically worsening under acyclovir and ceftriaxone, the hypothesis of autoimmune encephalitis was considered, and a cycle of IV immunoglobulin (0.4 g/kg/day for 5 days) was empirically started while waiting for specific antibody test results. A trial of Levetiracetam 1000 mg/day failed to improve myoclonic activity.

Considering the clinical picture of unspecific encephalopathy with an exuberant myoclonic state, we considered toxic aetiologies, namely bismuth encephalopathy, and again we questioned the patient’s relatives about the possibility of another medication other than the previously reported. The patient’s father reported chronic and irregular use of bismuth subsalicylate (Pepto-Bismol®), in tablets of 150 mg, for approximately 20 years, to treat gastroenterological complaints. The patient and her relatives had never considered this drug to be medically relevant, omitting it when initially asked about medication. The patient obtained the drug from the United States of America as it is not available in Portugal.

The bismuth level in the patient’s urine was 375 μg/L (normal < 3.0 μg/L), bismuth serum levels were 260 μg/L (normal < 0.5 μg/L), and bismuth levels in CSF were 21.4 μg/L (normal < 2.0 μg/L). These concentrations were measured eight days after admission, and all the results were highly above the laboratory’s threshold for bismuth toxicity.

Serum and CSF autoimmune encephalitis panel did not reveal the presence of abnormal antibodies. Negative microbiological CSF results (including PCR for HSV and CSF cultures) prompted acyclovir and ceftriaxone discontinuation. Supportive treatment was continued, and after two weeks we verified clinical improvement. She became more alert and aware, the myoclonic jerks became more subtle, and she performed autonomous gait. One month after admission, the level of bismuth in urine had reduced to 33.0 μg/L (normal < 3.0 μg/L), and the serum level reduced to 13.1 μg/L (normal < 0.5 μg/L). At the time of discharge, the patient scored 26/30 points in MoCA, and her mental and functional status continued to improve. On the 3-month follow-up, the patient was in her usual state of health, returning to work, without insomnia or other complaints. No alteration was documented on the neurological examination, and she scored 29/30 in MoCA test. The greyish discoloration of the teeth was less pronounced (a classical gingival bismuth line was never observed). EEG performed at that time was within the norms.

## Discussion and conclusions

This case highlights the fact that bismuth encephalopathy, while rare, should be considered in patients with subacute progressive encephalopathy, even after a chronic exposure. Recognition of bismuth encephalopathy was not straightforward mainly due to the rarity of this entity and the fact that encephalopathies of other aetiologies may present with a similar neurologic picture and CSF findings. Ali F. et al. [7] suggest a list of differential diagnoses: Creutzfeldt-Jakob disease and other neurodegenerative etiologies, other toxic/metabolic encephalopathy, neoplasms, infectious causes, vascular disorders, and systemic autoimmune disease with nervous system involvement. Although classically associated with a subacute clinical presentation, bismuth neurotoxicity can also manifest acutely. Brigandì A. et al. [8] reported a recent case of sudden onset neurological signs mimicking a stroke caused by bismuth subcitrate toxicity.

In addition to the diagnostic difficulties associated with a rare cause of encephalopathy, the patient and her family members initially omitted medication with bismuth, because they considered bismuth subsalicylate an innocuous drug, leading us to consider other causes such as infectious and autoimmune encephalitis. Only when we considered the hypothesis of bismuth encephalopathy, based on the absence of clinical response to antiviral and immune therapy and the presence of exuberant myoclonus, in a patient with a prior medical history of irritable bowel syndrome, did they report the use of this drug.

Bismuth subsalicylate (Pepto-Bismol®), marketed in the United States but not in Portugal, is an over-the-counter medication readily available via the internet, commonly used in the treatment of various gastrointestinal conditions, including dyspepsia, acute diarrhoea and in the prevention of traveller’s diarrhoea [9, 10]. In the age of online supplement use, with easy access to these substances, it is not clear that the general public or health care providers are fully aware that bismuth compounds can be toxic [5].

Since its initial development in 1918, Pepto-Bismol has become a widely known drug. Between 1973 and 1980, about 1000 cases of bismuth-related neurotoxicity were reported in France (among 942 patients, there were 72 deaths) [11], 40 in Australia and 26 in Belgium, Switzerland, and Spain [12]. These patients had ingested large doses of bismuth subnitrate or subgallate for long periods. (from 4 weeks to 30 years). However, only few of these cases has been published. Since the epidemic in France and Australia, a few other cases of bismuth-related neurotoxicity have been published, despite the continued use of bismuth salts worldwide [12, 13]. It is clearly established that many different types of bismuth preparation have caused the syndrome [13]. Neurotoxicity from use of topical bismuth dressing for burns also has been reported [14]. Previous authors observed similar symptoms in nine case reports of bismuth subsalicylate induced encephalopathy, summarized in Table 1.

**Table 1:** Summary of encephalopathy cases associated with bismuth subsalicylate

References	Age (years)	Sex	Presentations	Investigation	Outcome
Hasking GD. et al. (1982) [15]	60	M	Use of bismuth subsalicylate over some years. Dysarthria, ataxia, incontinence, and twitches.	Hight bismuth levels in urine and serum.	Full recovery in moths.
Mendelowitz PC. et al. (1990) [16]	45	M	7 days of bismuth large doses. Lethargy, dysarthria and myoclonic jerking that progressed to coma.	Hight bismuth levels in urine and serum. CT scan unremarkable.	Death.
Jungreis AC, et al. (1993) [17]	68	F	2 years of bismuth use. Tremor, myoclonic jerks, unsteady gait, confusion.	High serum level 6 weeks after stopping.	Recovery in 6 months.
Gordon MF. et al. (1995) [18]	54	M	Use of bismuth subsalicylate intermittently over many years. 6-week history of progressive confusion and a 2–3-week history of marked multifocal myoclonic jerks, postural tremors, postural instability, and gait ataxia.	Hight bismuth levels in urine and serum. EEG with bihemispheric slowing. MRI unremarkable.	Gradual recovery. Bismuth levels normalized after 12 weeks.
Reynolds PT. et al. (2012) [19]	56	F	2 moths of bismuth use. Psychomotor retardation, poor concentration, drowsy, visual hallucinations, tremor, myoclonic jerks and postural instability.	Hight bismuth levels in urine and serum. Slightly elevated white blood cell count in CSF. MRI unremarkable.	Full recovery in several months.
Masannat Y. et al. (2013) [20]	56	F	Weeks of bismuth use. Progressive confusion for two weeks, followed by myoclonus, tremors, gait instability and visual hallucinations.	Hight bismuth levels in urine and serum. CT scan, MRI, EEG and CSF were unremarkable.	Full recovery 4 months post discharge.
Siram R. et al. (2017) [21]	25	F	Hight bismuth doses for 15–20 days. Tinnitus, hearing loss (likely concurrent salicylate toxicity), encephalopathy, ataxia and myoclonus.	Hight serum bismuth levels. MRI with thalamic and basal ganglia signal changes, cerebral and cerebellar atrophy.	Improved over 2 months with residual hearing loss, cerebellar signs and dystonia.
Ali F. et al. (2017) [7]	54	F	2 years of bismuth use. 3-month history of progressive dementia, myoclonus, and ataxia	Hight bismuth levels in urine and serum. FDG-PET brain with diffuse mildly reduced uptake.	Marked recovery after withdrawal.
Hogan DB. et al. (2018) [6]	77	F	Long-term and uninterrupted consumption of bismuth subsalicylate. Impaired balance and gait, tremors and cognitive deficits.	Hight urine bismuth levels.	Marked recovery 1 month after withdrawal.

M: Male; F: Female

Our patient was going through a moment of intense stress and we know that psychological stress may have a critical effect on the gut-brain axis. Irritable Bowel Syndrome is considered a stress sensitive disorder. The effects of stress are mainly on intestinal motility, permeability, visceral sensitivity, immune responses and gut microbiota composition [22]. After oral administration, bismuth subsalicylate is nearly completely hydrolyzed in the gastrointestinal tract into bismuth and salicylic acid. Less than 1% of the bismuth in this compound is absorbed [12]. Cases of bismuth subsalicylate neurotoxicity reported have been marked by chronic overexposure, high-dose, or both. [6]. Why the disease appears so suddenly in patients with a chronic consumption is a question that cannot be fully answered satisfactorily [13]. Abnormal gut permeability (leading to increased absorption) and renal impairment (with decreased elimination) may predispose to bismuth toxicity [6]. Another theory is that this is not a toxic phenomenon directly proportional to bismuth consumption. Some authors believe in a modification of the intestinal microorganism that can convert the bismuth salt into an absorbable form, perhaps by methylation, and bismuth administered as an insoluble substance would become soluble and pass through the bloodbrain barrier [13, 23]. In our case, we hypothesized that a period of stress in a patient with Irritable Bowel Syndrome might have increased bismuth absorption and/or have changed the gut microbiota composition contributing to the neurotoxicity.

Subacute encephalopathy usually begins with a prodromal phase, which progresses over a few weeks or months, characterized by nonspecific cognitive and neuropsychiatric symptoms such as changes of mood and sleep, followed by a rapid deterioration over days with altered mental status, myoclonic jerks and ataxia [17, 19]. In our patient, insomnia lasted for years and we believe that it could be, at least in part, attributed to bismuth intake, mainly because it improved substantially after bismuth interruption.

Chronic use of bismuth-containing products can result in encephalopathy, whereas acute toxicity can manifest as nephrotoxicity [24]. Blackening of the tongue and teeth has been reported as a harmless side effect of bismuth toxicity [25]. Our patient had dark teeth, with no change in tongue coloration and no evidence of gingival bismuth line (Fig. 1). This discoloration of teeth, not so evident as previously described, probably should also be considered as an adverse effect of bismuth.

The levels of bismuth in urine, blood and CSF samples confirm the intoxication by this agent [5, 13]. Brain CT scan and MRI were both normal. Most of the other reported cases also didn’t show imaging changes [7, 16, 18–20]. However, Siram R. et al. [21] described a case of 25-year-old women who developed bismuth encephalopathy after taking high doses of bismuth subsalicylate for 15–20 days. Brain MRI showed signal changes in the dorsomedial thalamus and a repeated MRI, three months later, showed cerebral and cerebellar atrophy. Imaging changes result of the accumulation of bismuth in gray matter with edema in the surrounding white matter [21]. Buge A. et al. [26] also described a radiographic pattern of CT scan showing heterogeneous diffuse areas of cortical hyperdensity.

Treatment of bismuth encephalopathy consists of interruption of the offender along with supportive care. By removing bismuth-containing products, bismuth encephalopathy generally improves progressively over weeks to months [19]. Our patient returned three months after discharge without neurological symptoms. The role of chelators such as D,L-2,3-dimercaptopropane-1-sulfonic acid and 2,3-dimercapto-succinic acid is not well established [27, 28].

In conclusion, our case highlights that toxic encephalopathy should be considered when dealing with subacute encephalopathy. Bismuth encephalopathy may be difficult to recognize due to its rarity, infrequent documentation in the literature and because this drug is not considered medically relevant and therefore omitted by patients and relatives. Even when exposure to neurotoxins is not reported, the hypothesis of toxic encephalopathy should remain present and potential neurotoxic agent intake should be sought after. Chronic use of bismuth can cause neurotoxicity with devastating consequences and; however, its discontinuation is associated with a full recovery in weeks to months.

## Data Availability

All data and material supporting our findings are contained within the manuscript.

## Abbreviations

CSF: Cerebrospinal fluid
CT: Computed Tomography
EEG: Electroencephalogram
GP: General Physician
HIV: Human Immunodeficiency Virus
HSV: Herpes Simplex Virus
IV: Intravenous
MoCA: Montreal Cognitive Assessment
MRI: Magnetic Resonance Imaging
PCR: Polymerase Chain Reaction
